# Sucrose and invertases, a part of the plant defense response to the biotic stresses

**DOI:** 10.3389/fpls.2014.00293

**Published:** 2014-06-23

**Authors:** Alexandra S. Tauzin, Thierry Giardina

**Affiliations:** CNRS, Centrale Marseille, iSm2 UMR 7313, Aix Marseille UniversitéMarseille, France

**Keywords:** sucrose, cell wall invertase, vacuolar invertase, alkaline/neutral invertase, plant defense response

## Abstract

Sucrose is the main form of assimilated carbon which is produced during photosynthesis and then transported from source to sink tissues *via* the phloem. This disaccharide is known to have important roles as signaling molecule and it is involved in many metabolic processes in plants. Essential for plant growth and development, sucrose is engaged in plant defense by activating plant immune responses against pathogens. During infection, pathogens reallocate the plant sugars for their own needs forcing the plants to modify their sugar content and triggering their defense responses. Among enzymes that hydrolyze sucrose and alter carbohydrate partitioning, invertases have been reported to be affected during plant-pathogen interactions. Recent highlights on the role of invertases in the establishment of plant defense responses suggest a more complex regulation of sugar signaling in plant-pathogen interaction.

## Introduction

Cash and subsistence crops are susceptible to a large number of diseases caused by plant pathogens. Among pathogenic organisms: fungi, oomycetes, viruses and bacteria are the most important ones. The direct consequence of pathogen attack is the decrease of the crop yield. In addition to economic loss, consumer health may be compromised due to risks in ingesting toxins produced from secondary metabolites of these pathogens. Mycotoxins are probably the most known factors produced by fungi, which are not only poisonous but also carcinogenic for human (Maresca, 2013).

The plant response is mediated by a sophisticated immune system divided into two different pathways. The first is microbial-associated molecular-patterns-triggered immunity (MTI), constituted by elicitors recognized by the plant innate immune systems *via* pattern recognition receptors (PRRs) (Ausubel, 2005; Katagiri and Tsuda, 2010). The second is the effector-triggered immunity (ETI) stimulated on the basis of the perception of pathogen effectors by plant disease resistance proteins (Dangl and Jones, 2001; Jones and Dangl, 2006).

Pathogens modify the host metabolism which results in an energy increase and production of carbon sources (Thines et al., 2000) including sucrose and its cleavage products, glucose and fructose (Roitsch and Gonzalez, 2004; Rolland et al., 2006). Sucrose hydrolysis is catalyzed by invertases, and the consequence is the shifts of the apoplastic sucrose/hexose ratio in favor of hexoses. The aim of this paper is to review recent evidence on the crucial roles of invertases during plant pathogen attacks and how the invertase activity is regulated.

## From carbohydrate partitioning to plant defense response

### Sucrose signal molecule

In higher plants, sucrose is the major transport form of carbohydrates. Sucrose is produced during photosynthesis in source tissues (leaves), and then transported *via* the phloem to the different sink tissues (roots, stem, reproductive organs and vegetative storage organs) to provide the carbon and energy needed for growth and synthesis of storage reserves.

The role of sucrose as signaling molecule is well established (for reviews see Koch, 2004; Rolland et al., 2006; Wind et al., 2010; Tognetti et al., 2013). It affects plant development processes such as plant growth, regulation of flowering, differentiation of vascular tissue and development of storage organs (for review see Tognetti et al., 2013). Sucrose cleavage products, glucose and fructose, also act as signaling molecules. Of the two hexoses, glucose has been better described in relation with the hexokinase signaling pathway (Moore et al., 2003; Cho et al., 2009) while for fructose a specific pathway has been proposed involving the abscisic acid (ABA)- and ethylene-signaling pathway (Cho and Yoo, 2011; Li et al., 2011).

Gomez-Ariza et al. (2007) observed that the pre-treatment of rice plants with sucrose drastically reduced symptoms of fungal *Magnaporthe oryzae* infection and they proposed sucrose as a signal molecule in plant immunity.

### Plant invertases

Invertases (EC.3.2.1.26) hydrolyze irreversibly sucrose into glucose and fructose. Three groups were identified: alkaline/neutral invertases (A/NInv) localized in the cytosol, mitochondria and/or in plastids, and two types of acid invertases, insoluble bound to the cell wall (cell wall invertase, CWI) and soluble found in the vacuole space (vacuolar invertase, VI), respectively.

### Acid invertases and proteinaceous inhibitors

Acid invertases, CWIs and VIs, belong to the GH32 family. CWIs play a key role in sucrose partitioning, plant development and cell differentiation while VIs are involved in cell expansion, sugar storage and regulation of cold induced sweetening (Roitsch and Gonzalez, 2004). Both are post-translationally regulated by proteinaceous inhibitors (INHs) which belong, with pectin methylesterase inhibitors (PMEIs), to the pectin methylesterase inhibitor related protein (PMEI-RP) family (Pfam 04043) (Hothorn et al., 2004).

During plant infection, the level of VI modulation is poorly understood with contradictory reports in the literature that leads to an unclear functional assignment (Table 1). On the one hand, a reduction of VI expression has been observed during the infection of *Vicia faba* by *Uromyces fabae* and *Vitis vinifera* by *Erysiphe necator* and *Plasmopora viticola* (Voegele et al., 2006; Hayes et al., 2010). This down-regulation was attributed to a decrease in the availability of sucrose in the storage compartment (Voegele et al., 2006; Hayes et al., 2010). By contrast, a high VI activity was observed during the first stage of infection of castor beans by *Agrobacterium tumefaciens* that might suggest a supportive function during invasion (Wachter et al., 2003). Moreover, the expression of a VI (TIV-1) is not affected in tomato infected by *Botrytis cinerea* (Hyun et al., 2011). Finally, when Essmann et al. compared wild type tobacco plants and transgenic plants silenced for CWI after infection by *Phytophthora nicotianae*, they noticed no significant changes in the VI activity (Essmann et al., 2008a,b) suggesting that the VI is not involved in the plant defense response. These results reinforce the doubts concerning the exact role of VIs in plant immunity.

**Table 1:** **Summary of plant pathogen interaction studies referring to invertase modulations**.

**Microorganism**	**Plant**	**Effects on invertase**	**Additional features**	**References**
**BACTERIA**				
*Erwinia carotovora*	Carrot	CWI (+)	Induction of *PAL*	Sturm and Chrispeels, 1990
*Agrobacterium tumefaciens*	*Ricinus communis*	CWI (+) VI (+)	Change in sugar content, ABA synthesis	Wachter et al., 2003
*Xanthomonas campestris* pv *vesicatoria*	Tomato	CWI (+)	Change in sugar content, induction of senescence-associated and *PR* genes	Kocal et al., 2008
*Xanthomonas campestris* pv *vesicatoria*	Pepper	CWI (+)	Induction of defense response *PR-Q*	Sonnewald et al., 2012
Bois noir	Grapevine	CWI (+)	Callose deposition, modulation of *SUC* genes	Santi et al., 2013a,b
*Xanthomonas oryzae* pv. *oryzae*	Rice	CWI (+)	Change in sugar content, callose deposition, induction of *PR* genes, ROS accumulation	Sun et al., 2013
**FUNGI**				
**Biotrophic**				
*Erysiphe pisi*	*Pisum sativum*	CWI/VI (+) A/NInv (+)	Decrease of starch content	Storr and Hall, 1992
*Puccinia hordei*	Barley	CWI/VI (+)	ND	Tetlow and Farrar, 1992
*Blumeria graminis*	Barley	CWI (+) VI (+)	Change in sugar content, down-regulation of photosynthesis, callose deposition, induction of defense response *PR-1*	Scholes et al., 1994; Wright et al., 1995; Swarbrick et al., 2006
*Blumeria graminis*	Wheat	CWI (+) VI (+)	ND	Greenshields et al., 2004
*Blumeria graminis*	Wheat	CWI (+) VI (+) A/NInv (+)	Change in sugar content	Sutton et al., 2007
*Albugo candida*	*A. thaliana*	CWI (+) VI (/)	Change in sugar content, decrease of starch content, down-regulation of photosynthesis, decrease chloropyll content, induction of defense proteins	Chou et al., 2000
*Erysiphe cichoracearum*	*A. thaliana*	CWI (+)	Induction of *HXT* genes	Fotopoulos et al., 2003
*Uromyces fabae*	*Vicia faba*	CWI (+) VI (−)	ND	Voegele et al., 2006
*Erysiphe necator*	*Vitis vinifera*	CWI (+) VI (−)	Induction of *HXT* and ABA biosynthesis-associated genes	Hayes et al., 2010
**Hemibiotrophic**				
*Magnaporthe grisea*	Rice	CWI (+)	Change in sugar content, callose deposition, induction of *PR* genes, ROS accumulation	Cho et al., 2005; Sun et al., 2013
**Necrotrophic**				
*Fusarium oxysporum*	Tomato	CWI (+)	ND	Benhamou et al., 1991
*Botrytis cinerea*	Tomato	CWI (+) VI (/)	ND	Hyun et al., 2011
**Symbiotic**				
*Glomus intraradices*	Tomato	CWI (+)	ND	Schaarschmidt et al., 2006
*Glomus intraradices*	Tobacco	CWI (+)	Change in sugar content, exchange of nutrients, decrease chloropyll content, induction of *PR* genes	Schaarschmidt et al., 2007
**OOMYCETES**				
*Phytophthora nicotianae*	Tobacco	CWI (+) VI (/) A/NInv (+)	Down-regulation of photosynthesis, callose deposition, induction of *PR* and *PAL* genes	Scharte et al., 2005; Essmann et al., 2008a,b
*Plasmopara viticola*	*Vitis vinifera*	CWI (+)	Induction of *HXT* and ABA biosynthesis-associated genes	Hayes et al., 2010
**RHIZARIA**				
*Plasmodiophora brassicae*	*A. thaliana*	CWI (+) VI (+)	ND	Siemens et al., 2011
**NEMATODE**				
*Heterodera schachtii*	*A. thaliana*	CWI (−) VI (−) A/NInv (−)	Change in sugar content	Cabello et al., 2013
*Meloidogyne javanica*	*A. thaliana*	CWI (−) VI (−) A/NInv (+/−)	Change in sugar content	
**VIRUS**				
Potato virus Y	Tobacco	CWI (+) VI (/)	Down-regulation of photosynthesis, induction of *PR* genes, callose deposition	Herbers et al., 2000
Beet severe curly top virus	*A. thaliana*	CWI (+)	Callus-like structures, induction cell cycle-related genes	Park et al., 2013

Abbreviations: (+), up-regulation; (−), down-regulation; (/), no change; ABA, abscisic acid; HXT, hexose transporter; PR, pathogenesis-related; ROS, reactive oxygen species; SUC, sucrose transporter; ND, not described.

By contrast, the link between plant response against pathogen and CWI activity has been widely studied (Table 1). A common trend is observed for the rapid increase of the CWI mRNA level after infection by bacterial, fungal, viruses, oomycetes and nematodes (for detailed references see Table 1). Indeed, the up-regulation of CWI activity is essential to modulate sugar partitioning and provide the sugars which are necessary for the pathogen development. A clear example has been demonstrated for gall development in *A. thaliana* (Siemens et al., 2011). Moreover, it was shown that during infection CWI activity also triggers plant defense responses such as induction of defense-related gene expression, callose deposition and reduction of photosynthesis or cell death. CWI silencing disrupts the ability of transgenic plants to answer correctly to the pathogen attacks and impairs the defense induced reaction (Essmann et al., 2008a). In rice, the loss-of-function mutant of the CWI gene GRAIN INCOMPLETE FILLING 1 (GIF1) has been demonstrated to be hypersusceptible to postharvest pathogens while the constitutive expression of GIF1 enhances the resistance to pathogens by activating the plant defense response (Sun et al., 2013). In the particular case of symbiosis (such as arbuscular mycorrhiza), the expression of CWI is finely controlled by the partner to prevent the induction of *pathogenesis-related* (PR) genes and promote “long-term” interaction (Schaarschmidt et al., 2006, 2007).

Invertase activity is potentially modulated by proteinaceous inhibitors (INHs) in a pH-dependent manner (Tauzin et al., 2014). Greiner et al. (1998) demonstrated that tobacco INH didn't affect invertases purified from two fungi, *Candida utilis* and *Saccharomyces cerevisiae*, supporting the idea that INHs are not involved in plant defense mechanisms. However, a strong repression of the expression of one of the three INHs from *A. thaliana* after infection by *Pseudomonas syringae* pv. tomato DC3000 was documented (Bonfig et al., 2010). The invertase activity was detectable only in infected plants while the enzyme was present in infected and uninfected crude extract cells, indicating that the enzyme activity was repressed by a specific inhibitor. This result was corroborated by the utilization of the pseudo tetrasaccharide acarbose which inhibits invertase activity *in planta* resulting in an increased susceptibility of the infected plant compared to the wild type (Bonfig et al., 2010).

### Alkaline/neutral invertases

A/NInvs are non-glycosylated proteins and they belong to the GH100 family (Lammens et al., 2009). They have different subcellular localizations such as cytosol, mitochondria, chloroplast and nuclei (Vargas and Salerno, 2010). A/NInvs are involved in plant growth and development, flowering and seed germination (Jia et al., 2008; Barratt et al., 2009; Welham et al., 2009). Xiang et al. (2011) demonstrated that A/NInvs are part of the antioxidant system involved in cellular reactive oxygen species homeostasis. Moreover, exogenous application of gibberellic acid (GA) rescued the delay of germination in the seeds of the A/NInv mutants suggesting a communication between A/NInv and phytohormones (Xiang et al., 2011; Martin et al., 2013).

Correlated with the increase of the CWI activity, an increase of the A/NInv activity has been observed in *Pisum sativum*, tobacco and *A. thaliana* during infection by powdery mildew (Storr and Hall, 1992), oomycetes (Essmann et al., 2008a), and the beet curly top virus (Park et al., 2013), respectively. Interestingly, in transgenic tobacco plants silenced for CWI, the A/NInv activity remained unchanged during the interaction with the oomycetic phytopathogen (Essmann et al., 2008a). The authors suggested that the CWI activity increased first and by consequence the availability of carbohydrate changes and triggers the A/NInvs activity as a secondary phenomenon in the plant immunity (Essmann et al., 2008a). By contrast, the infections of *A. thaliana* by two different nematodes *Heterodera schachtii* and *Meloidogyne javanica* led to the down-regulation of *A/NInv* gene (*AtCINV1*) reflected by a decrease of activity (Cabello et al., 2013). Thus, the importance of A/NInv might vary depending on the pathosystem.

## Defense-induced features affected by sucrose and invertases

### Clock, photosynthesis, and sugar content

The connections between the clock, the sugars and the immunity have been previously presented (Roden and Ingle, 2009; Bolouri Moghaddam and Van Den Ende, 2013) and here we discuss the latest updates on this interconnectivity. Exogenous sucrose is able to stimulate the circadian clock by inhibiting photosynthesis and to coordinate answers during the light-dark cycles (Knight et al., 2008; Dalchau et al., 2011; Haydon et al., 2013). A new metabolic feedback loop involving the morning-expressed *pseudo response regulator 7* (*prr7*) gene was proposed by Haydon et al. (2013). At dawn, the light activates PRR7 and photosynthesis, then the photosynthetically produced derived sugars accumulate and repress the PRR7 promoter which causes the de-repression of the molecular oscillator component circadian clock associated 1 (CCA1) (Haydon et al., 2013). The clock-related genes (*cca1* and *lhy*) affect stomatal aperture after pathogen infection and suggest a crucial role of circadian clock in plant defense response (Wang et al., 2011; Zhang et al., 2013). Diurnal rhythm has been shown to regulate a CWI (LIN6) from tomato and that both CCA1 and LHY activate the *Lin6* promoter (Proels and Roitsch, 2009). During pathogen attack, the increase of CWI activity leading to an accumulation of hexoses is associated with a down-regulation of photosynthesis and expression of genes-related to photosynthesis (Table 1). It is noteworthy that transgenic infected plants silenced for CWI showed a delay in the reduction of photosynthesis (Kocal et al., 2008). Thus, the cross-talk between clock, sucrose and invertases tends to illustrate that a fine regulation of the sucrose/hexose ratio is crucial in defense regulation (Haydon et al., 2013).

During the day, both sucrose and starch are produced during photosynthesis. During the night, the starch, accumulated in the chloroplasts, is subsequently degraded to provide substrates for sucrose synthesis. Starch synthesis can be regulated by sucrose and clock by modulating the expression of starch synthase (Wang et al., 2001). After pathogen infection, a decrease in the starch content is observed in the infected region suggesting that the degradation of starch provides more substrates to sucrose synthesis. Interestingly, Engelsdorf et al. tested the susceptibility of starch-free *A. thaliana* mutants against biotrophic, hemibiotrophic and necrotrophic pathogens and pointed out that depending on the studied pathosystem the diurnal carbon availability is a susceptibility factor (Engelsdorf et al., 2013). Their results imply that sugar availability might impact the ability of plants to trigger defense responses.

One of the other possibilities for changing the sugar content is the regulation of the expression of the sucrose transporter. Sucrose acts on carbohydrate partitioning and phloem loading by modulating the sucrose transporter expression, such as inducing the expression of *SUT2* in tomatoes or repressing the expression of *BvSUT1* in beet (Barker et al., 2000; Vaughn et al., 2002). Depending on the stage of infection, the expression of sucrose transporters can be altered and as a consequence the sucrose partitioning can be modified. In rice infected by *Xanthomonas oryzae* pv. *Oryzae*, SWEET proteins are upregulated and sucrose accumulates in apoplast ready to be used for the pathogen growth (Chen et al., 2010, 2012). Santi et al. reported a sequential regulation of sucrose transporter genes which are first downregulated during infection of grapevine by stolbur to limit the spread and then upregulated during the recovery stage providing necessary nutrients (Santi et al., 2013a,b). It is noteworthy that during fungal infection the expression of CWI and hexose transporters displayed a correlation enhancing the hexoses supply from the phloem to the surrounding tissues during the transition from source to sink (Fotopoulos et al., 2003; Hayes et al., 2010). Moreover, Hayes et al. reported a relationship between CWI, hexose transporters and ABA biosynthesis during the transition from source to sink after infection (Hayes et al., 2010).

### Phytohormones

For different phytohormones such as ABA, gibberellins, ethylene and jasmonate, it was shown that they interact with the sucrose signaling pathway (Finkelstein et al., 2002; Leon and Sheen, 2003; Gibson, 2004; Heil et al., 2012). Their implication in plant defense response and the relationship with sugars have been widely discussed in various reviews (Bolouri Moghaddam and Van Den Ende, 2012, 2013).

### Pathogenesis related proteins

PR proteins are synthesized in response to plant pathogen attack. Their classification and their properties have been well described (for reviews see Kitajima and Sato, 1999; Van Loon et al., 2006; Sels et al., 2008). As reported in several studies, the up-regulation of CWI due to the infection goes along with the induction of *PR* genes (Table 1) such as PR-1a, PR-1b, PR3, PR10, WRKY45, and NPR1 in rice (Sun et al., 2013), PR-1b and PR-Q in tobacco (Herbers et al., 1996; Schaarschmidt et al., 2007; Essmann et al., 2008b) and PR-Q, Pin-II and GluB in tomato (Kocal et al., 2008). During transgenic approaches the overexpression of CWI in tobacco or in rice presented constitutively high levels of *PR* transcripts compared to the wild type plants (Herbers et al., 1996; Sun et al., 2013). To support this idea, in different cases of infected transgenic plants silenced for CWI, the induction of *PR* genes was abolished (Schaarschmidt et al., 2007; Essmann et al., 2008b; Kocal et al., 2008). Thus CWI activity is required to enhance the expression of *PR* genes mediated by the accumulated hexoses which act as signal molecules. Besides, exogenous sucrose induced the expression of *PR* genes (Thibaud et al., 2004; Gomez-Ariza et al., 2007) confirming the idea of sucrose as an important signal molecule for plant defense response.

### Phenylpropanoid pathway

The phenylalanine ammonia-lyase (PAL), a key enzyme which is involved in the phenylpropanoid pathway, leads to the biosynthesis of lignin and the production of many other important compounds such as the flavonoids, coumarins and lignans (for review see Dixon and Paiva, 1995). During infection of lupine by *Fusarium oxysporum*, sucrose induced the phenylpropanoid metabolism by stimulating the activity of PAL (Morkunas et al., 2005, 2011). Sturm and Chrispeels showed an accumulation of PAL mRNA subsequently to the increase of CWI mRNA in carrot infected by *Erwinia carotovora* (Sturm and Chrispeels, 1990). Moreover when tobacco plants are silenced for CWI, the PAL activity is delayed after infection compared with the wild type plants (Essmann et al., 2008b). Hence, the regulation of PAL is mediated by the variation of the sucrose/hexose ratio. All in all, these results demonstrate that the regulation of the expression of PAL is sugar-related.

Anthocyanin (a flavonoid) has an antimicrobial potential reducing the spread of the pathogens. The synthesis of anthocyanin is regulated by sucrose signaling pathway (Solfanelli et al., 2006) through the induction of the *PAP1/MYB75* transcription factor (Teng et al., 2005) and ABA and jasmonate pathways have a synergic effect (Loreti et al., 2008). This induction is repressed by gibberellins. At concentrations of sucrose higher than 2% the anthocyanin synthesis is induced independently of the ABA signaling pathway (Dai et al., 2014). Recently, a key positive regulator in the sucrose signaling pathway controlling the anthocyanin synthesis has been identified as the DELLA protein which targets *PAP1/MYB75* (Li et al., 2014).

In potato tubers, a transcription factor (*AN1*) was proposed to up-regulate the phenylpropanoid pathway. The authors suggested that *PAL* might be induced by *AN1* after sucrose feeding. Moreover they proposed a loop in which sucrose increases *AN1* expression while *AN1* induces sucrolytic enzymes which release hexoses used by the phenylpropanoid pathway (Payyavula et al., 2013). By the synthesis of secondary metabolites such as phenolic compounds or later on lignin, plants produce chemical and physical barriers against pathogens.

### Cell wall reinforcement

As another physical barrier, there is the deposition of callose, a β -(1,3)-glucan cell wall polymer, which is a stress related process limiting invasion by regulating the plasmodesmata and the sieve plates permeability (Chen and Kim, 2009; Luna et al., 2011). In tobacco plants overexpressing a yeast invertase in the apoplast or in the vacuole, the increase of callose deposition was comparable to that observed in wild type plants infected with potato virus Y (Herbers et al., 1996). These results were consistent with a positive regulation of callose deposition by GIF1 in rice after infection by both, bacterial and fungal pathogens (Sun et al., 2013), leading to a regulation mediated by CWI activity. Increasing concentrations of the exogenous sucrose repressed the callose deposition in *A. thaliana* cells (Luna et al., 2011) suggesting that hexose cleavage products of sucrose are responsible for the formation of the physical barrier against invading pathogens through cell wall reinforcement.

## Conclusion and perspectives

Due to a high demand in carbohydrates during infection, plants evolved strategies to modulate their carbohydrate availability and trigger to defense responses. In most of the studied pathosystems, sucrose seems to act as a “priming” agent activating a cascade of signaling pathways such as the modulation of circadian clock genes, phytohormones, cell wall strength and cellular signaling pathways.

A rapid induction of CWIs after infection increases the hexose content and modulates sink strength. It has been demonstrated that CWIs are essential for triggering an appropriate answer during pathogen invasion. The accumulation of hexoses leads to an induction of the *PR* genes, a down-regulation of the photosynthesis, and an establishment of the chemical and physical barriers. A/NInvs, which are induced afterwards, might be involved in providing more energy during infection. The exact function of the VIs remains unclear but they might release stored carbohydrates and allow reserves mobilization. Moreover, the specificity of plant response depending on the studied pathosystem might be interesting points to investigate.

A better understanding of the “sweet immunity” and the complex network between sucrose, circadian clock and phytohormones might be useful to avoid substantial losses in yield and quality of crops every year. Recently, these biotic elicitors were proposed as interesting elements to generate ready-to-eat cruciferous vegetables and maximize their health-promoting compounds (Baenas et al., 2014).

## Author contributions

Alexandra S. Tauzin and Thierry Giardina contribute equally to the writing of this review.

### Conflict of interest statement

The authors declare that the research was conducted in the absence of any commercial or financial relationships that could be construed as a potential conflict of interest.
